# Utilizing Medical Student Feedback to Improve Teaching Cases in Pre-clerkship Curricula

**DOI:** 10.1007/s40670-024-02128-3

**Published:** 2024-08-02

**Authors:** Lia Pierson Bruner, Leah Topper, Amy Baldwin, Ellen M. House, M. Tresa Chappell, Janette R. Hill

**Affiliations:** 1 Department of Family and Community Medicine, Augusta University/University of Georgia Medical Partnership, UGA Health Sciences Campus, Russell Hall, Room 235K, 1425 Prince Ave., Athens, GA 30602 USA; 2 Department of Family Medicine and Community Health, Duke University School of Medicine, Durham, NC USA; 3 Department of Biochemistry and Molecular Biology, Augusta University/University of Georgia Medical Partnership, Athens, GA USA; 4 School of Social Work, University of Georgia, Athens, GA USA; 5 Department of Psychiatry and Health Behavior, Augusta University/University of Georgia Medical Partnership, Athens, GA USA; 6 Department of Pediatrics, Augusta University/University of Georgia Medical Partnership, Athens, GA USA; 7 Learning, Design, & Technology, University of Georgia, Athens, GA USA

**Keywords:** Student-directed learning, Active learning, Curriculum development, Educational scholarship, Pre-clinical medical education

## Abstract

Feedback is an essential part of continuous quality improvement of cases used in medical curricula. This report describes results of qualitative analysis of feedback to elucidate what worked well and what needed improvement from the lens of our pre-clerkship medical students. Complexity, realism, and use of media were themes identified as strengths. Increasing the complexity, realism, and media as well as clarifying test results were themes for suggested improvements. While some feedback themes were similar across pre-clerkship years, others differed between first- and second-year students, likely representing the evolution of our learners.

## Background

Patient cases are important and widely used in healthcare education [1]. Within undergraduate medical education, cases are an integral aspect of case-based learning (CBL) and problem-based (PBL) modalities. CBL, an example of active learning, has been shown to have benefits over standard lecture and team-based learning [1–5]. Many medical schools have adopted some form of CBL in their curricula and face the challenge of developing, maintaining, and updating their own teaching cases. Resources have been published and can help guide the process of developing and evaluating individual cases [6, 7]. There is also guidance for improving diversity, inclusion, and equity in curricular materials in medical education [8–13]. Core attributes of effective individual cases have been identified as relevant, realistic, engaging, challenging, and instructional [6, 7]. When individual cases are combined to create a case catalog, desired attributes should be expanded to include being consistent, current, diverse and inclusive, patient-centered, and mission-centered [8].

While the overall effectiveness of PBL and CBL as learning modalities has been studied, limited data are available on the effectiveness of the cases themselves, which can take a variety of forms and be used differently at each institution [1–5]. Similarly, most published student feedback on CBL appears to be focused on the overall CBL experience rather than the content or quality of the teaching cases [14]. The University of Ottawa used medical student perspectives of the positives and negatives of their cases to develop a detailed tool for creating and reviewing CBL cases, focused primarily on case structure and inclusion of factual information on pathophysiology, differential diagnosis, and treatment [7]. As many medical schools move to integrate more clinical aspects into pre-clerkship curricula, student perspectives on effectiveness of CBL cases that include communication skills, ethics, professionalism, and person-centered care are important.

At our institution, faculty have worked to integrate clinical and foundational science content of our pre-clerkship medical curricula that emphasizes student-directed, case-based small groups that work through one case weekly in year 1 and two cases weekly in year 2. The small groups usually consist of eight medical students and two faculty facilitators, typically a physician and a basic scientist. The groups meet for 2-h sessions three times per week to go through cases, generate learning agendas, share student learning summaries, and take part in case presentations and case role plays. During the COVID-19 pandemic, there were no changes to the structure or process of these groups other than meeting virtually.

Given the centrality of the cases to our curriculum, a case oversight team was established in 2018 to design and implement a case catalog quality improvement process, a key component of which was the collection and use of student feedback [8]. We initially prioritized humanizing case patients, increasing case patient diversity, and updating diagnosis and treatment details. We also made the cases more complex and realistic with increased coverage of social determinants of health, mental health concerns, ethical and professionalism topics, and modeling of communication and patient education. Additionally, we increased coverage of primary care scenarios, supporting the mission of our institution to produce more primary care doctors for our state. We report here the thematic analysis of qualitative case feedback obtained from multiple cohorts of medical students to identify themes for high-quality CBL cases to improve learning and guide systematic improvements in a pre-clerkship case catalog.

## Activity

Student feedback on patient cases used in the pre-clerkship curricula is an important source of information for case revision at our institution. Student groups complete an online form at the end of each week where they answer two questions about each case: “Which aspects of the case most effectively facilitated your learning, and why?” and “Which aspects of the case could be improved?”.

To identify themes, we first compiled the weekly, anonymous, free-text student feedback from late spring 2018 through December 2021. The overall group feedback response rate was 89% across the 206 weekly opportunities for group feedback over the study period. Our campus class size increased from 40 to 60 students during this time, so feedback was solicited from the five to eight groups in each of the M1 and M2 classes, each composed of seven to nine students. These data were organized by each academic year and student cohorts (i.e., year 1 and year 2). Two team members used an inductive analysis approach to develop 535 in vivo codes, combine the initial codes into 30 broader categories, and then re-code data using the categories to generate themes. When representing the voices of participants is important, using an inductive, constant-comparative approach for data analysis is suggested [15, 16]. This ensured that the perspectives of the students were fully represented in the presentation of the themes. Themes regarding the aspects of the cases that facilitated their learning and aspects that could be improved were identified within years 1 and 2, and then across both years 1 and 2.

## Results and Discussion

**Fig. 1:**
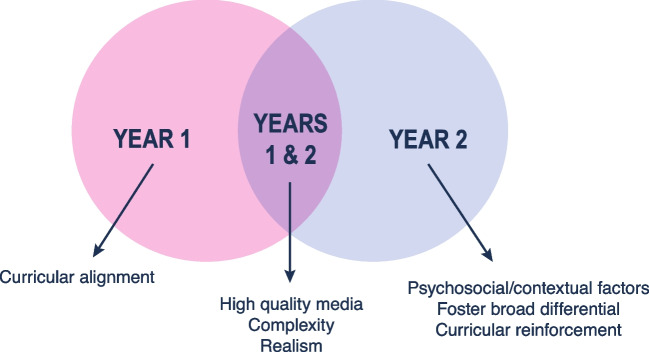
Case aspects that facilitated learning

Pre-clerkship student feedback across years 1 and 2 revealed three leading characteristics of cases that facilitated learning (Fig. 1): inclusion of high-quality media (e.g., images, audio, video), complexity with rich details, and realism. In addition, year 1 students appreciated when the cases aligned with other curricular components (e.g., gross anatomy, clinical skills), and year 2 students valued incorporating psychosocial/contextual factors (e.g., ethics, social determinants), fostering the development of broad differentials, and curricular reinforcement.

**Fig. 2:**
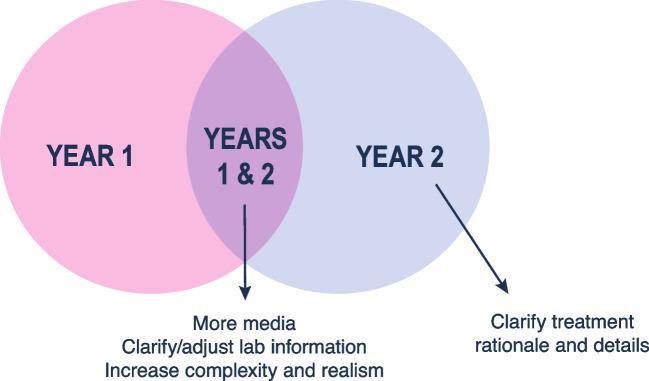
Case aspects for improvement

Themes for suggested improvement from both years (Fig. 2) included requests for more media, clarifying/adjusting lab information, and more details to increase complexity and realism. Year 2 students also requested clarification of treatment rationale and details. Example quotes illustrating the themes are provided in Table 1.

**Table 1:** Sample quotes illustrating case themes

Case aspects that facilitated learning	Sample student quotes
Inclusion of high-quality media	“The imaging was very helpful for understanding the progression of the disease.” “This was our first pediatrics case… and the video demonstrating breathing retractions on physical exam was helpful.”
Complexity with rich details	“Having two patients with different pathologies helped facilitate the learning. There are various types of brachial plexus injuries, and it was interesting to compare and contrast them. The multigenerational aspect was a nice aspect. It was realistic that Mr. Gonzalez may have sought medical help after seeing similar symptoms in his grandson.”
Realism	“The case… was relevant to recent events with Covid-19, had a more realistic feel, incorporated complexity of the patient's conditions (i.e., progression of complications), and was relevant to our position as medical students.”
Curricular alignment	“Having the case correlated directly to a lecture was helpful because it allowed time to think and ask questions outside of group. Having a unique dynamic with a family member that has issues as well was useful and accurate.”
Incorporation of psychosocial/contextual factors	“We appreciated the inclusion of social determinants of health which facilitated important discussion on behalf of the healthcare provider.”
Foster broad differential	“Made you consider a large differential… [and] realistic in terms of loss of follow up, [and] ethical/ prognostic factors of chemotherapy.”
Curricular reinforcement	“Great broad case that covered a lot of material from this week… and some from previous weeks. Great revisiting shock and ARDS. Having to think about the DDx when considering her occupation (nurse) and the etiology of [pneumonia].”
Case aspects for improvement	
Inclusion of more media	“Would be nice to see CXR from Wednesday… also an EKG of atrial fibrillation. More pictures please i.e., radiographs, pictures of clinical manifestations.”
Clarification/adjustment of lab information	“Panel of lab result values to interpret would be useful.” “Would have been nice to have lipase for pancreatic function.”
Inclusion of more details to increase complexity and realism	“Could be made more complicated by having Sjogrens.” “Could be more realistic in terms of the logistics/difficulties of her initial hospitalization.”

It is striking that the three themes of realism, complexity, and media appeared in both the aspects that facilitated learning as well as areas for improvement. Rather than representing contradictory feedback from different groups, it appears from the specific wording of the quotes that students both appreciated these aspects and requested an increase in quality and quantity. Furthermore, while students earlier in their pre-clerkship training highlighted curricular alignment as important, year 2 students appreciated reinforcement of previous material, accurate clinical management, inclusion of psychosocial/contextual factors, and cases that fostered broad differentials. These differing priorities may reflect the development of the learners throughout the pre-clerkship curriculum.

The importance of realism, complexity, and media in curricular cases were themes in our study and are consistent with existing literature that has identified authenticity, realism, being “engaging and challenging” (i.e., complexity), and use of media as important components of effective case-based learning cases [6, 14, 17, 18]. Although person-centered, diverse, and inclusive attributes have been described as important aspects of CBL cases and case catalogs, it is unclear how commonly these factors are included in standard CBL cases [8]. For our students, inclusion of psychosocial and contextual factors was another positive theme, especially in year 2. In addition, this study documented changes to student priorities as they progressed through the pre-clerkship curriculum with increased interest in differentials, psychosocial factors, treatment, and curricular reinforcement. Careful crafting of cases can provide students with complex and diverse scenarios in which to practice contextualizing pathophysiology and clinical management in the setting of realistic biopsychosocial circumstances.

### Strengths and Limitations

A strength of this study was analysis of extensive data including multiple years of free-text feedback on over 100 individual cases from students at different stages of pre-clerkship training. Another strength was the consistency between coders working with a robust code set to generate themes. The findings reported in this study, as well as the process for obtaining and incorporating feedback, may be applied to other health professions’ case-based curricula at various levels of training.

As our cases are updated each year for continuous quality improvement, one limitation of this study is that it is not possible to directly compare identical cases across years. Therefore, feedback likely changed in response to case revisions, and extracted themes may have evolved.

A next step includes analyzing feedback for trends over time. Because each class of students only experiences one version of the cases, future directions include using focus groups of students and faculty to compare different versions of the same cases to more directly assess perceptions of case modifications on learning and preparation for clerkships.

## Conclusions

To improve learning outcomes, it is important to understand which aspects of curricular cases are highly valued by medical students. This study has demonstrated that increased realism, complexity, and media were strong themes for our pre-clerkship learners. Based on the results of this study, educators could consider a review of cases in their curriculum to ensure they contain valued attributes and are aligned with the curriculum. Medical educators may also consider implementing a process for collecting student feedback to guide systematic case improvements and development of future cases, with an overall goal of promoting the growth of well-rounded physicians who are better prepared to deliver patient-centered care.

## Data Availability

Data available upon request.
